# Childhood visual impairment and health-related quality of life in Ghana: evidence from a cross-sectional study

**DOI:** 10.1186/s12955-026-02565-1

**Published:** 2026-06-27

**Authors:** Nana Akwasi Owusu Mensah, Josephine Ampomah Boateng, Isaiah Osei Duah Junior, Albert Kwadjo Amoah Andoh, Godsway Commanda Osei, Sylvester Kyeremeh, Sylvia Agyekum, Gabriel Kwaku Agbeshie, Werner Eisenbarth, Kwadwo Owusu Akuffo

**Affiliations:** 1 Department of Optometry and Visual Science, College of Science, Kwame Nkrumah University of Science and Technology, Kumasi, Ghana; 2 Department of Psychology, John R. and Kathy R. Hairston College of Health and Human Sciences, North Carolina Agricultural and Technical State University, Greensboro, NC 27411 USA; 3 Department of Applied Science and Mechatronics, HM Hochschule München University of Applied Sciences, Munich, Germany

**Keywords:** Vision loss, Health-related quality of life, Pediatric Quality of Life Inventory, Ghana

## Abstract

**Background:**

Although visual impairment (VI) is known to affect children’s development, there is limited evidence on how VI influences health-related quality of life (HRQoL) among children in Ghana. This study aimed to assess the relationship between childhood VI and HRQoL.

**Methods:**

A cross-sectional study was conducted among children aged 2 to 18 years in selected districts of the Ashanti Region, Ghana. Comprehensive ocular assessments including visual acuity (VA), refraction, and anterior and posterior segment evaluations were performed. Health-related quality of life was measured using the PedsQL 4.0 across physical, emotional, social, and school functioning domains. Data were analyzed using descriptive statistics, independent-sample t-tests, and linear regression.

**Results:**

A total of 581 children participated (mean ± SD age: 8.64 ± 4.00 years), with females representing 53.0% of the sample. Both child-reported and parent-reported scores showed significantly reduced HRQoL among children with VI across all PedsQL domains compared with children without VI, with school (*p* < 0.001) and emotional (*p* = 0.005) functions of child-reported showing statistical significance. Presenting VI was a significant predictor of poor overall HRQoL in both simple (β= -10.351, *p* < 0.001) and multiple linear regression models (β= -5.650, *p* = 0.016). No significant associations (p > 0.05) were found between HRQoL and sociodemographic variables, including sex, age, ethnicity, work status, and health insurance.

**Conclusions:**

Childhood VI is associated with substantial reductions in multiple dimensions of HRQoL. These findings underscore the need to strengthen early detection, clinical management, and school-based vision support services to improve pediatric eye care in Ghana.

## Introduction

Childhood visual impairment (VI) is a major global health concern with substantial negative impact on optimal functioning and development [1, 2]. According to the World Health Organization, an estimated 19 million children under 15 years live with VI, of whom 1.4 million are irreversibly blind [3, 4]. The burden is disproportionately higher in low- and middle-income countries, where prevalence of childhood blindness ranges from 0.2 to 7.8 per 10,000 children [5–7]. For instance, Africa alone accounts for 19% of global blindness, underscoring its disproportionate responsibility for VI and blindness [8]. A systematic review further reported that the age-standardized prevalence of blindness in the region is the highest among all Global Burden of Disease super-regions and nearly double the global average [9, 10]. Evidence from a rural African population shows that the duration and severity of vision loss substantially affect vision-related quality of life (QoL) [11]. In Ghana, Amedo and colleagues [12] similarly reported lower QoL scores across physical, social, psychological, and environmental domains among individuals attending a low-vision clinic. Despite growing global evidence linking childhood VI with reduced health-related quality of life (HRQoL), there remains a paucity of evidence from Ghana. This evidence gap, necessities a region-specific evaluation of pediatric HRQoL assessment to inform screening strategies and health intervention policies to optimize children vision care in Ghana.

QoL broadly encompasses subjective well-being as well as objective aspects such as self-actualization and cultural integration [13], spanning physical, mental, and social dimensions of health [14]. More specifically, HRQoL is shaped by three interrelated components: functional capacity, personal health perceptions, and symptom status [15, 16]. These components align with the physical, social, emotional and school domains that are directly influenced by a person’s health, reflecting the lived experiences of children with VI. In pediatric populations, validated QoL assessment tools offer insight into the burden of VI and its broader impact on health outcomes [17–19]. Studies consistently show that children with VI experience reduced HRQoL across multiple domains, highlighting the need for targeted interventions [20–24]. Such children frequently face academic challenges, reduced independence, and social exclusion [21], as well as increased risk of neurodevelopmental and psychomotor delays, especially when visual problems begin early in life [25–27]. These challenges may accumulate over time, affecting both the child and family. In contexts such as Ghana, where specialized pediatric eye care and inclusive educational services are limited, the effects are even more pronounced, and unmet need for low-vision care remains high [28]. While clinical evaluation can quantify the degree of visual loss, HRQoL assessments provide a more holistic understanding of its real-life impact [29–31], guiding individualized management strategies [12]. For pediatric care, such assessments help clinicians tailor interventions that support not only visual function but also developmental, educational, and psychosocial needs.

This study therefore aimed to evaluate the HRQoL of children with and without VI in the Ashanti region of Ghana. Specifically, it sought to answer: *What differences exist in HRQoL outcomes between children with and without VI? Which HRQoL domains are mostly affected by VI?* This aligns with the Sustainable Development Goals (SDGs), particularly SDG 3, which emphasizes ensuring healthy lives and promoting well-being for all at all ages [32]. Reducing avoidable childhood blindness and improving rehabilitation services supports broader goals related to education, productivity, and social inclusion. Given the substantial number of life-years affected by pediatric VI, understanding how visual limitations influence physical, emotional, social, and functional well-being is essential. Identifying the most affected domains can guide targeted interventions and inform policies that integrate eye health into child health and education programs, contributing meaningfully to the SDG agenda.

## Methods

### Study design, population, and setting

This cross-sectional study was carried out among children living in selected districts of the Ashanti region, Ghana’s second most densely populated region according to the 2021 Population and Housing Census. Districts were stratified into four categories based on shared demographic characteristics, including level of urbanization, literacy rates, economic activities, and population density as defined by the Ghana Statistical Service (2021a). The four resulting strata were: (1) Highly Urbanized and Densely Populated Districts, (2) Peri-Urban, Medium-Populated, Rapidly Developing Districts, (3) Balanced Rural–Urban, Medium-Populated Districts, and (4) Rural and Less Densely Populated Districts. From each stratum, one district was purposively selected, guided by child population size and approximate gender balance. The selected districts were Afigya Kwabre South (Peri-Urban, Medium-Populated, Rapidly Developing), Oforikrom Municipal (Highly Urbanized and Densely Populated), Atwima Kwanwoma (Balanced Rural–Urban, Medium-Populated), and Bosome Freho (Rural and Less Densely Populated). Because 61.6% of the Ashanti Region’s population resides in urban and peri-urban areas, an additional district—Juaben Municipal, classified as Peri-Urban, Medium-Populated, Rapidly Developing—was randomly selected from these categories to improve representativeness.

## Study participants and eligibility criteria

Child inclusion criteria: Children aged 2 to 18 years were included. VI was defined as a presenting visual acuity (VA) of 6/18 or worse, in accordance with the International Classification of Diseases, 11th Revision (ICD-11) [33]. The comparison group had no VI (VA better than 6/18).

Parent/guardian definition: A primary caregiver (biological parent or legal guardian or foster parent or relative) who lives with the child and was responsible for daily care. The parent/guardian completed the parent-perceived report for the child in the study.

Exclusion criteria were children with significant comorbid conditions known to independently affect HRQoL. These children were excluded to minimize confounding and to isolate the impact of VI on HRQoL.

### Sampling and sample size

A stratified purposive technique was employed to select study centers and recruit study participants. A minimum sample size of 384 was calculated using the Cochran’s formula applicable for cross-sectional studies (𝑛=𝑍^2^⋅𝑝(1−𝑝)/𝑒^2^), where n is the initial sample size, Z is the 1.96 for a 95% confidence interval, p (set at 0.5) is the estimated proportion of the characteristics of interest and e is the margin of error. The proportion p was set at 0.5 as the most conservative estimate to maximize sample size, and the margin of error was set at 0.05. To account for a non-response rate of 5%, the target sample size was adjusted to 404 participants.

### Data collection procedures

Demographic information including age, sex, parent or guardian education, ethnicity, occupation, and district was collected for all participants. Each child underwent a comprehensive eye examination conducted by two licensed optometrists and trained optometry student volunteers. Examinations included VA testing, cover testing, autorefraction, and anterior and posterior segment evaluation. Entrance and best-corrected VA were assessed at both distance and near to determine the child’s visual recognition threshold, using Teller acuity [34] and Lea grating paddles. The VA measurements were recorded in Logarithm of the Minimum Angle of Resolution (LogMAR) units for analysis. Refractive status was objectively measured using the Plus Optix Autorefractor A12R, while anterior and posterior segment evaluations were performed in a dimly lit room using an ophthalmoscope. Children identified with VI were referred to the KNUST University Hospital Eye Clinic for follow-up examination and appropriate management.

### Quality of life assessment

The Pediatric Quality of Life Inventory (PedsQL™) [35, 36] was used to assess outcomes. The PedsQL is a validated instrument for measuring HRQoL in healthy children and adolescents as well as those with acute and chronic conditions. Its measurement model integrates generic core scales and disease-specific modules into a single system, allowing for comprehensive evaluation. PedsQL questionnaire has been utilized in some research studies in Ghana [37–39]. This confirms the questionnaire’s practical applicability in the Ghanaian setting. Even though some global studies [21, 40–43] have reported its reliability and validity in assessing HRQoL in pediatric populations, there are currently no such studies conducted in Ghana. In this study, the PedsQL™ 4.0 Generic Core Scales, consisting of 23 items across four domains (Physical, Emotional, Social, and School Functioning), were administered to children recruited from vision screening in the selected districts to assess QoL outcomes. Each item is rated on a 5-point scale (0 = never a problem, 1 = almost never a problem, 2 = sometimes a problem, 3 = often a problem, 4 = almost always a problem). For ease of interpretation, responses were reverse-scored and linearly transformed to a 0–100 scale (0 = 100, 1 = 75, 2 = 50, 3 = 25, 4 = 0), with higher scores indicating better HRQoL and lower scores indicating reduced HRQoL or greater impairment.

All children who completed the visual assessment went ahead with the HRQoL assessment. The PedsQL was administered according to the participant’s age to ensure developmental appropriateness and accurate reporting. Parents and guardians (a primary caregiver responsive for the child’s daily care) also completed the parent-report questionnaires. Children aged 2–4 years completed the Parent Report for Toddlers [36], while children aged 5–7 years completed the Parent and Child Report [44]. For participants aged 8–12 years, both the Parent Report and Child Report were used. Adolescents aged 13–18 years completed the Teen Report along with the Parent Report for Teens [45]. All instruments were validated for pediatric populations and administered in a standardized format to ensure consistency and reliability. Questionnaires were completed on the same day, using interviewer administration, with field staff assisting children and guardians as needed. Field staff were trained optometry students and licensed optometrists who volunteered. Field staff read the questions, clarified items where necessary and recorded participant responses for consistency. For participants who were not fluent in English, the questionnaire was administered in the local language (Twi) through interviewer-assisted translation. Where necessary, items were translated verbally and explained by trained field staff to ensure participants’ understanding. No formal validated translated version was used.

### Ethical approval

The study was performed in accordance with the Declaration of Helsinki and ethical approval was obtained from the Committee on Human Research, Publication and Ethics (CHRPE), Kwame Nkrumah University of Science and Technology, Kumasi, Ghana (CHRPE/AP/1245/24). Permission was sought from district health directorates and community heads. Written and informed consent was obtained from the study participants and their parents or guardians. Consent forms were available in both English and Twi, the predominant languages in the Ashanti region. In cases where necessary, the consent form was read out to the participant and their guardian.

### Statistical analysis

Data were analyzed using IBM SPSS Statistics version 27. Descriptive analyses were conducted by calculating frequencies and percentages for categorical variables, and means and standard deviations (SD) for continuous variables. PedsQL scores, originally recorded on a 5-point Likert scale, were reverse-scored and linearly transformed to a 0–100 scale for analysis. Domain and total scores were computed as the mean of non-missing items. If more than 50% of items in a domain were missing, the domain score was not computed and treated as missing and excluded. Differences in demographic characteristics between children with and without VI were compared using chi-square test. An independent sample t-test was used to compare PedsQL domain scores between children with and without VI. Where Levene’s test for equality of variances was significant, Welch correction (equal variances not assumed) was used. Internal consistency and reliability of the PedsQL 4.0 subscales were assessed using Cronbach’s α. Simple and multiple linear regression analyses were performed to identify demographic factors associated with HRQoL while adjusting for potential confounders. Demographics were selected based on established literature, geographic variation in healthcare and indicators of health. Variables with p-values less than 0.20 in simple regression analyses were included in the multiple regression models. This threshold was employed to avoid omitting potentially important predictors.

## Results

### Description of the sample

A total of 581 participants were included in the study, comprising 273 males (47.0%) and 308 females (53.0%). The majority of participants were aged 8–12 years (41.3%), followed by those aged 5–7 years (21.2%) and 2–4 years (18.8%). Most guardians identified as Akan (78.3%), with smaller proportions identifying as Ewe (4.1%), Mamprusi (3.3%), Kusasi (3.4%), Frafra (2.1%), and other ethnicities (8.8%). In terms of education, nearly half (48.4%) of guardians had completed high school, while 29.4% had primary education, 12.0% had no formal education, and 9.6% had tertiary education. The majority were employed (84.5%), with 12.7% unemployed and 2.2% retired. Most participants (91.9%) had no presenting distance VI, while 3.3% were identified with it. The largest proportion of participants came from Oforikrom Municipal (25.8%), followed by Afigya Kwabre South (23.1%), Atwima Kwanwoma (22.2%), and Bosome Freho and Juaben Municipal (each 14.5%). Regarding the children’s education, more than half (55.8%) were in primary school, 25.0% in preschool, 15.7% in secondary school, and 3.6% had no formal education. Most were full-term births (77.6%), while 2.4% were preterm. A large majority (89.5%) were covered by health insurance, and 10.2% were not covered by health insurance as shown in Table 1.

**Table 1:** Description of the sample

Variable	*n* (%)
**Total**	581(100)
Sex	
Male	273 (47.0)
Female	308 (53.0)
**Age group in** **years **	
2–4	109 (18.8)
5–7	123 (21.2)
8–12	240 (41.3)
13–18	106 (18.8)
**Districts**	
Afigya Kwabre South	134 (23.1)
Oforikrom Municipal	150 (25.8)
Atwima Kwanwoma	129 (22.2)
Bosome Freho	84 (14.5)
Juaben Municipal	84 (14.5)
**Child’s educational level**	
Pre-School	145 (25.0)
Primary	324 (55.8)
Secondary	91(15.7)
No formal education	21 (3.6)
**Birth term**	
Full term	451 (77.6)
Pre term	14 (2.4)
Missing	116 (20.0)
**Child health insurance**	
Yes*	520 (89.5)
No*	59 (10.2)
NA	2 (0.3)
**Presenting distance VI**	
No	534 (91.9)
Yes	19 (3.3)
**Guardians Ethnicity**	
Akan	455 (78.3)
Ewe	24 (4.1)
Mamprusi	19 (3.3)
Frafra	12 (2.1)
Kusasi	20 (3.4%)
Others	51 (8.8)
**Guardian education level**	
No formal education	70 (12.0)
Primary	171(29.4)
High School	281(48.4)
Tertiary	56 (9.6)
Missing	3 (0.5)
**Guardian Work status**	
Employed	491(84.5)
Unemployed	74 (12.7)
Retired	13 (2.2)
Missing	3 (0.5)

Yes= With presenting distance VI; No= Without presenting distance VI

During analysis, 28 participants were exempted due to their inability to co-operate during the VA assessment. In the analysis of demographic characteristics between the two groups (children with and without VI), there was no significant difference in sex distribution (*p* = 0.686), ethnicity (*p* = 0.361), guardian level of education (*p* = 0.580), guardian work status (*p* = 0.159) district (*p* = 0.363), birth term (*p* = 0.367) and child’s health insurance (*p* = 0.708) amongst the two groups. In contrast, age distribution (*p* < 0.001) and child’s educational level (*p* < 0.001) showed significant difference between the two groups as shown in Table 2.

**Table 2:** Demographic characteristics between children with and without VI

Variable	WITH VI n(%)	WITHOUT VI n (%)	*p*-value
**Total (n = 553)**	19 (3.4)	534 (96.6)	
Sex			0.686
Male	8 (42.1)	250 (46.8)	
Female	11(57.9)	284 (53.2)	
**Age** ** group in years **			< 0.001
2–4	10 (52.6)	73 (13.7)	
5–7	2 (10.5)	120 (22.5)	
8–12	3 (15.8)	236 (44.2)	
13–18	4 (21.1)	105 (19.7)	
**Districts**			0.363
Afigya Kwabre South	4 (21.1)	124 (23.2)	
Oforikrom Municipal	6 (31.6)	138 (25.8)	
Atwima Kwanwoma	6 (31.6)	118 (22.1)	
Bosome Freho	0 (0.0)	78 (14.6)	
Juaben Municipal	3 (15.8)	76 (14.2)	
**Child’s educational level**			< 0.001
Pre-School	9 (47.4)	117 (21.9)	
Primary	4 (21.1)	319 (59.7)	
Secondary	2 (10.5)	89 (16.7)	
No formal education	4 (21.1)	9 (1.7)	
**Birth term**			0.367
Full term	13 (92.9)	415 (97.0)	
Pre term	1(7.1)	13 (3.0)	
**Child’s health insurance**			0.708
No	2 (10.5)	52 (9.8)	
Yes	17 (89.5)	480 (90.2)	
**Guardians Ethnicity**			0.361
Akan	15 (78.9)	423 (79.8)	
Ewe	1(5.3)	22 (4.2)	
Mamprusi	2 (10.5)	16 (3.0)	
Frafra	0 (0.0)	11(2.1)	
Kusasi	0 (0.0)	17 (3.2)	
Others	1(5.3)	45 (7.7)	
**Guardian’s education level**			0.580
No formal education	1(5.3)	65 (12.2)	
Primary	4 (21.1)	163 (30.7)	
High School	12 (63.2)	250 (47.1)	
Tertiary	2 (10.5)	53 (10.0)	
**Guardian Work status**			0.159
Employed	14 (73.7)	453 (85.3)	
Unemployed	4 (21.1)	67 (12.6)	
Retired	1(5.3)	11(2.1)	

*28 participants were excluded because of failed cooperation during visual acuity assessment

### Internal consistency reliability

The internal consistency of the PedsQL 4.0 subscales was evaluated using Cronbach’s alpha (α), with values above 0.70 generally considered acceptable for research purposes. For child-reported outcomes, mean subscale scores ranged from 84.28 (emotional function) to 93.51 (social function). Cronbach’s alpha values ranged from 0.54 (social function) to 0.73 (physical function). The overall reliability for child self-report was α = 0.79 (standardized α = 0.81). For parent-reported outcomes, mean scores were slightly higher across domains (range: 87.75–96.30). Reliability coefficients ranged from 0.56 (emotional function) to 0.64 (physical function), with standardized alpha’s improving to 0.59–0.77. The overall parent-reported reliability was strong (α = 0.80; standardized α = 0.85). Overall, both child and parent reports of the PedsQL 4.0 demonstrated acceptable to good reliability, with parent-reported scales showing slightly higher internal consistency compared to child reports (see Table 3). Agreement between parent-proxy and child self-reported PedsQL total scores was assessed using the intraclass correlation coefficient (ICC) with a two-way mixed-effects model for absolute agreement. A moderate level of agreement was observed (ICC = 0.675, 95% CI: 0.530–0.767, *p* < 0.001).

**Table 3:** Internal consistency and reliability of PedsQL 4.0 subscales

PedsQL 4.0 Subscales	Mean	Cronbach’s alpha (α)	Cronbach’s alpha based on standardized items
**Child reported (5–18 years)**			
Physical function	89.85	0.70	0.73
Emotional function	84.28	0.63	0.64
Social function	93.51	0.51	0.64
School/Work function	85.10	0.54	0.54
Overall	88.37	0.79	0.81
**Parent reported (2–18 years)**			
Physical function	91.34	0.64	0.74
Emotional function	88.57	0.56	0.59
Social function	96.30	0.63	0.77
School function	87.75	0.62	0.62
Overall	91.08	0.80	0.85

PedsQL=Pediatric Quality of Life Inventory

### Quality of life

Table 4 presents the mean scores and standard deviations for the PedsQL 4.0 subscales across different age groups. Children’s self-reported PedsQL scores showed consistently lower QoL among those with VI compared with peers without VI across all age groups. Among the 5–7 years age group, children with VI reported the lowest scores in the physical function domain (59.38 ± 22.10 vs. 89.29 ± 12.21). For the 8–12 years age group, children with VI demonstrated substantially poorer scores across all domains, with the lowest scores in the school function domain as compared to those without VI (60.00 ± 14.14 vs. 85.58 ± 14.38). Likewise, adolescents aged 13–18 years, with VI reported the least scores in the school functioning domain as compared with those without VI(61.25 ± 24.62 vs. 85.10 ± 14.20). Parent-reported assessment scores, reflected a similar pattern in reduced school functioning among wards with VI. For children aged 2–4 years, differences between VI and non-VI groups were minimal across all domains. Emotional function domain recorded the lowest scores (65.00 ± 7.07) in children with VI in the 5–7 years age group, school function domain had the least scores (57.50 ± 3.54) in the 8–12 years age group, and the least scores (73.75 ± 27.50) in the 13–18 years age group. (see Table 4).

**Table 4:** PedsQL subscale scores across different age groups

Age group	Domain	VI present (mean ± SD)	VI absent (mean ± SD)	Total (mean ± SD)
**Child Reported**				
5–7 (*n* = 119)				
	Physical Function	59.38 ± 22.10	89.29 ± 12.21	88.54 ± 13.00
	Emotional Function	80.00 ± 14.14	88.97 ± 17.09	88.93 ± 16.90
	Social Function	90.00 ± 0.00	96.03 ± 8.60	95.73 ± 8.68
	School Function	75.00 ± 21.21	85.85 ± 16.23	85.70 ± 16.18
8–12 (*n* = 228)				
	Physical Function	76.56 ± 15.47	91.21 ± 12.75	90.64 ± 13.70
	Emotional Function	65.00 ± 28.28	83.12 ± 17.72	82.94 ± 17.62
	Social Function	72.50 ± 3.54	93.74 ± 11.35	93.35 ± 12.06
	School Function	60.00 ± 14.14	85.58 ± 14.38	85.36 ± 14.61
13–18 (*n* = 103)				
	Physical Function	72.66 ± 22.44	90.45 ± 11.61	89.61 ± 12.32
	Emotional Function	63.75 ± 14.93	83.73 ± 17.90	81.97 ± 18.32
	Social Function	72.50 ± 37.75	92.25 ± 11.24	91.35 ± 13.16
	School Function	61.25 ± 24.62	85.10 ± 14.20	83.84 ± 15.58
**Parent Reported**				
2–4 (*n* = 90)	Physical Function	83.44 ± 8.34	86.30 ± 11.91	84.52 ± 12.57
	Emotional Function	91.00 ± 11.25	84.19 ± 17.09	85.39 ± 15.68
	Social Function	97.50 ± 7.91	97.50 ± 8.02	97.91 ± 9.54
	School Function	88.54 ± 18.32	88.42 ± 17.23	85.81 ± 18.07
5–7 (*n* = 119)	Physical Function	68.75 ± 26.52	92.34 ± 9.47	91.67 ± 10.21
	Emotional Function	65.00 ± 7.07	91.14 ± 13.02	90.68 ± 13.26
	Social Function	95.00 ± 7.07	96.67 ± 6.16	96.59 ± 6.25
	School Function	82.50 ± 24.75	87.09 ± 14.83	87.15 ± 14.82
8–12 (*n* = 228)	Physical Function	87.50 ± 8.84	93.59 ± 10.72	93.29 ± 11.83
	Emotional Function	62.50 ± 53.03	88.54 ± 15.57	88.26 ± 16.07
	Social Function	67.50 ± 31.82	96.31 ± 8.62	95.92 ± 9.61
	School Function	57.50 ± 3.54	88.80 ± 14.12	88.51 ± 14.45
13–18 (*n* = 103)	Physical Function	82.03 ± 21.71	94.10 ± 10.02	93.57 ± 10.69
	Emotional Function	92.50 ± 15.00	90.20 ± 12.12	90.10 ± 12.27
	Social Function	75.00 ± 33.17	97.15 ± 8.95	96.13 ± 11.36
	School Function	73.75 ± 27.50	89.05 ± 13.67	88.46 ± 14.51

Children aged 2–4 only completed parent-reported PedsQL; Child reported – participant physical, social, emotional and school related quality of life functioning average in the past month; Parent reported- parent perceived physical, social, emotional and school related quality of life functioning average in the past month; VI- presenting VA 6/18 or worse; VI present= presenting VI; VI absent = no presenting VI.

Table 5 also presents results comparing PedsQL scores between children with and without VI with pooled data across all age groups (child-reported: 5–18 years; parent-perceived: 2–18 years). For child-reported outcomes, children with VI had significantly lower scores on emotional function (67.78 ± 16.03 vs. 84.57 ± 17.71, *p* = 0.005) and school function (64.38 ± 19.90 vs. 85.50 ± 14.94, *p* < 0.001) domains compared to children without VI. For parent-perceived outcomes, no domain reached statistical significance after Bonferroni correction (all *p* > 0.006). The largest difference was observed in physical function (77.96 ± 20.74 vs. 92.45 ± 10.63, *p* = 0.007).

**Table 5:** Comparison of PedsQL domain between children with and without visual impairment (pooled analysis)

Domain	VI present (Mean ± SD)	VI absent (Mean ± SD)	t-value	df	*p*-value*
Child-reported Pooled (5–18 years)					
Physical Function	63.88 ± 26.34	90.38 ± 12.35	3.01	8.07	0.017
Emotional Function	67.78 ± 16.03	84.57 ± 17.71	2.82	463	0.005
Social Function	71.67 ± 28.94	93.94 ± 10.68	2.31	8.04	0.050
School Function	64.38 ± 19.90	85.50 ± 14.94	3.94	460	< 0.001
Parent-perceived Pooled (2–18 years)					
Physical Function	77.96 ± 20.74	92.45 ± 10.63	3.03	18.34	0.007
Emotional Function	82.89 ± 20.09	88.97 ± 14.63	1.31	18.70	0.208
Social Function	86.84 ± 22.19	96.69 ± 8.06	1.93	18.17	0.069
School Function	79.69 ± 21.21	88.37 ± 14.59	1.63	15.45	0.124

* Bonferroni correction was applied thus only *p* < 0.006 is considered significant

Bivariate simple linear analyses (see Table 6, Model 1) showed that the variables significantly associated (*p* < 0.20) with HRQoL were age group, guardian educational level, presenting VI, birth term, child’s educational level, child’s health insurance and guardians’ work status. Model 2 adjusted for age group and sex only. In this model, VI remained strongly associated with lower HRQoL (β = -9.785, *p* < 0.001). On including the variables that were found to be significant in the linear regression model, the multiple regression analyses (F(14,420) = 3.828; R-squared = 0.113, *p* < 0.001) showed that, VI remained a strong and significant predictor of lower HRQoL (β = -5.650, *p* = 0.016). Children whose parents were unemployed (β = -2.813, *p* = 0.019) and children whose parents had no formal education (β = -6.138, *p* = 0.029) had significantly lower HRQoL scores compared to those with some education. Children born pre-term also had significantly lower HRQoL scores than full-term children (β = -7.406, *p* < 0.001).

**Table 6:** Linear regression assessing factors affecting HRQoL

	Simple regression			Multiple regression					
	Model 1			Model 2			Model 3		
	Unstandardized co-ef (β)	S.E	*p* value	Unstandardized co-ef (β)	S.E	*P* value	Unstandardized co-ef (β)	S.E	*P* value
**Sex**									
Male vs. Female	0.072	0.776	0.926	-0.140	0.756	0.853			
**Age group**									
2–4	Ref								
5–7	3.633	1.206	0.003	1.408	1.272	0.269	0.045	1.536	0.977
8–12	3.792	1.063	< 0.001	1.507	1.149	0.190	-0.652	1.826	0.721
13–18	4.355	1.253	< 0.001	2.350	1.306	0.072	1.698	2.319	0.464
**Guardian level of education**									
No formal education	Ref								
Primary	0.945	1.316	0.473				0.076	1.479	0.959
Secondary	-1.674	1.238	0.177				-0.781	1.382	0.572
Tertiary	-1.248	1.655	0.451				-2.813	1.741	0.375
**Presenting VI**									
No vs. Yes	-10.351	2.047	< 0.001	-9.785	2.094	< 0.001	-5.650	2.336	0.016
**Guardian work status**									
Employed	Ref								
Unemployed	-3.005	1.163	0.010				-2.813	1.190	0.019
Retired	-2.056	2.588	0.427				2.557	3.653	0.484
**Child health Insurance**									
No vs. Yes	2.199	1.289	0.089				2.048	1.343	0.128
**Child’s educational level**									
Pre-school	Ref								
Primary	2.854	0.912	0.002				2.142	1.458	0.143
Secondary	2.120	1.234	0.086				-0.426	2.116	0.841
No formal education	-5.585	2.122	0.009				-6.138	2.797	0.029
**Birth term**									
Full term vs. Preterm	-7.103	2.310	0.002				-7.406	2.187	< 0.001

F (14, 420) = 3.828; R-squared = 0.113, *p* < 0.001; S.E., Standard Error of Unstandardized co-efficient (β); Model 1(unadjusted); Model 2 (age and sex adjusted); Model 3 (adjusted for all significant variables); *p* < 0.20 considered statistically significant in simple linear regression

## Discussion

This study examined HRQoL outcomes among children with VI and explored factors influencing HRQoL using PedsQL 4.0 outcomes among a pediatric sample in Ghana [35]. Children with VI reported significantly lower HRQoL across all domains, particularly in school and emotional functioning, compared to children without VI. After adjusting for sociodemographic and clinical factors, presenting distance VI, and preterm birth were significantly associated with reduced HRQoL. Guardian’s educational level and work status also influenced HRQoL outcomes. These highlight the multifaceted impact of pediatric VI on physical, social, emotional, and school-related functioning.

Children in low-income settings are disproportionately affected by VI due to limited access to healthcare, nutritional deficiencies, and inadequate sanitation, further compounding its impact on their QoL [46]. These selective disadvantages often result in poorer educational outcomes, restricted social engagement, and a higher burden of mental health challenges, highlighting the need to understand their specific circumstances [47]. Similarly, VI in children does not directly affect their academic achievements and social interactions only but also has a significant impact on their caregivers, particularly parents, who often experience a reduced QoL due to the demands of caring for a child with a permanent disability [48–51]. To this end, this study hypothesized that children with VI in low-income settings, will experience lower HRQoL compared to their peers without VI, after adjusting for age and sex. This disparity is expected to manifest across physical, emotional, social, and school functioning, compounded by limited access to healthcare, socioeconomic challenges, and the additional burden on caregivers. Based on this premise, we examined differences in HRQoL outcomes between children with and without VI, while exploring the influence of preterm birth and demographic factors on these outcomes.

In this study, children with VI had lower PedsQL scores across physical, social, emotional, and school/work domains compared to peers without VI. This finding is consistent with previous research showing significantly lower HRQoL among children with VI, with strong correlations between HRQoL and both distance and near VA [20, 21, 52, 53]. Other studies have similarly reported mild to moderate impacts of VI on overall QoL [53]. This study further underscores these disparities by highlighting specific deficits in physical, emotional, social, and school functioning. VI can limit participation in physical play and sports, restrict safe navigation, and hinder engagement in classroom activities, thereby affecting physical competence and academic self-esteem [54, 55].

Lower emotional functioning scores likely reflect experiences of frustration, social anxiety, and withdrawal commonly reported among children with VI [56]. These findings emphasize the need for interventions that go beyond visual rehabilitation to also support psychosocial well-being. School-based programs, inclusive educational environments, and early psychosocial interventions can enhance emotional resilience, participation, and overall QoL among children with VI.

Our study also found children with VI had significantly lower HRQoL scores compared to their peers without VI after adjusting for age and sex. This supports our hypothesis and is consistent with existing literature [20, 57]. The magnitude of the effect observed in Model 2 of our multiple regression suggests the substantial impact of VI on HRQoL independent of demographic characteristics such as age and sex.

Children born preterm in this study also exhibited reduced HRQoL, consistent with evidence linking prematurity to long-term health and developmental challenges [58–60]. Preterm birth has been associated with reduced HRQoL, affecting physical function, daily activities, and family well-being [61–63], as well as an increased risk of abnormal visual and neurological development, which may further reduce vision-related QoL [58]. Collectively, these findings underscore the cumulative burden of prematurity and the importance of early, multidisciplinary interventions integrating ophthalmologic monitoring, neurodevelopmental care, and psychosocial support to improve long-term outcomes.

Our finding that guardian unemployment and lack of formal education predict lower HRQoL underscores the influence of socioeconomic disadvantage on pediatric health outcomes. Children whose guardians were unemployed had lower HRQoL scores, likely reflecting reduced household financial capacity that limit access to timely and appropriate healthcare services. Similarly, guardians with no formal education may face challenges in understanding and navigating health information, adhering to treatment recommendations and advocating for their child’s rehabilitative needs. These findings are consistent with previous studies [47, 64] reporting socio-economic status as key determinants of pediatric HRQoL.

Of note, the PedsQL instrument demonstrated acceptable psychometric performance in this study, with strong internal consistency for both child- and parent-reported outcomes (α = 0.79 and 0.80, respectively). Lower reliability for the social and school/work domains in child reports may reflect differences in how children interpret or prioritize these aspects of life, highlighting the need for age-appropriate and contextually sensitive HRQoL assessment tools in diverse pediatric populations. The generic PedsQL was used because of its global applicability and ability to allow direct comparability with other pediatric chronic eye disease population. Although it lacks vision-specific items, it captures domains of physical, emotional, social and school functioning relevant to children with chronic conditions including VI.

This study has several strengths. The participant sample was broadly representative of the pediatric population in Ghana, providing insights that reflect the general impact of VI on children’s QoL across the country. Our findings extend the literature by reporting child-reported and parent-perceived scores across all individual HRQoL domains using a standardized and validated instrument. Importantly, while the group of children with VI (*n* = 19) was relatively small, the inclusion of a larger comparison group of children without VI increased the robustness of the findings, allowing for reliable contrasts between affected and unaffected children. This design enabled detailed analysis of the multidimensional effects of VI and can inform policy development, as well as guide the planning of targeted rehabilitative and preventive services for affected children. However, the study also has some limitations. The cross-sectional design precludes causal inference between VI and reduced HRQoL. The relatively small number of children with VI limited the statistical power for some subgroup analyses and the unequal age distribution in group sizes could potentially influence the outcomes. Also, data on comorbid health conditions were not collected thus its effect on HRQoL could not be analyzed. Finally, the findings may not be generalizable to all pediatric populations in Ghana or other low-resource settings with differing sociocultural contexts.

## Conclusion

In conclusion, this study clearly demonstrates all HRQoL domains (physical, social, emotional and school functioning) are negatively affected by VI. Children born premature are likely to experience reduced HRQoL. Guardian unemployment and lack of formal education influence HRQoL, indicating that improving health outcomes requires addressing socioeconomic barriers alongside clinical care. These findings highlight the importance of early health screening, rehabilitative interventions, and educational and social support to improve the overall QoL in children. 

## Data Availability

The datasets generated and analyzed during the current study are not publicly available due to patient confidentiality but are available from the corresponding author on reasonable request.

## Abbreviations

QoL: Quality of Life
PedsQL: Pediatric Quality of Life Inventory
SSA: Sub-Saharan Africa
CHRPE: Committee on Human Research, Publication and Ethics
VI: Visual Impairment
HRQOL: Health-Related Quality of Life
VA: Visual Acuity
SDG: Sustainable Development Goals
